# Costs of non-metastatic prostate cancer treatment among privately insured men in the United States

**DOI:** 10.1371/journal.pone.0324902

**Published:** 2025-05-30

**Authors:** Ashley J. Housten, Su-Hsin Chang, Hannah E. Rice, Allison J. L’Hotta, Eric H. Kim, Bettina F. Drake, Joanna L. Buss, Mary C. Politi

**Affiliations:** 1 Department of Surgery, Division of Public Health Sciences, Washington University School of Medicine, St. Louis, Missouri, United States of America; 2 Department of Surgery, Department of Physiology and Cell Biology, University of Nevada Reno School of Medicine, Reno, Nevada, United States of America; 3 Department of Medicine, Institute for Informatics, Washington University School of Medicine, Saint Louis, Missouri, United States of America; 4 School of Public Health, Washington University in St. Louis, St. Louis, Missouri, United States of America; Indira Gandhi Medical College and Hospital, INDIA

## Abstract

**Background:**

Our objective was to quantify the cumulative total and out-of-pocket (OOP) costs for 3 non-metastatic prostate cancer treatment modalities: radiation, surgery, and conservative management at intervals of 1-, 3-, and 5-years post-diagnosis. We predicted these cumulative costs for a typical patient to improve cost transparency, facilitate conversations about potential costs, and to help advance non-metastatic prostate cancer cost evaluation.

**Methods:**

We used Merative™ MarketScan® Commercial Database data from 2007–2020. The cumulative total costs evaluated from the healthcare sector perspective were patient, clinician, and system/facility costs. We used descriptive statistics to summarize the sociodemographic characteristics of the cohort and a multivariable regression model to estimate the association between each treatment option (radiation, surgery, conservative management) and costs with inverse probability of treatment weighting (IPTW) to account for potential selection bias. We then predicted total and OOP costs defined by sample mode characteristics.

**Results:**

This cohort included 74,324 patients. Cumulative total and OOP costs were significantly higher for radiation (p < 0.0001) and for surgery (p < 0.0001) at Years 1, 3, and 5 compared to conservative management. For a typical patient, total cumulative cost estimates for conservative management at Years 1/3/5 were: $15,896/$33,436/$48,110 and the cumulative patient OOP costs were: $2,003/$4,540/$6,621. The cumulative total costs for surgery at Years 1/3/5 were: $38,348/$49,424/$60,885 and the cumulative OOP costs were: $2,980/$5,255/$7,221. The cumulative total costs for radiation at Years 1/3/5 were: $65,397/$77,859/$91,497 and the cumulative OOP costs were: $3,151/$5,481/$7,504.

**Conclusions:**

For all years, the cumulative costs of radiation were highest, followed by surgery and conservative management, respectively. Radiation as the first treatment modality had higher costs compared to surgery and conservative management at the 3 time points.

**Impact:**

These cost estimates support non-metastatic prostate cancer treatment related cost transparency. These estimates can help researchers evaluate costs and facilitate patient-clinician cost conversations.

## Introduction

Financial harm, including financial toxicity, creates substantial  burden among those undergoing cancer treatment [1]. Financial harm is common throughout cancer treatment and extends into survivorship contributing to disparities and lower quality-of-life [1]. TIndividuals experiencing financial harm are less likely to adhere to their medication, more likely to forgo filling prescription drugs, and less likely to follow-up with needed medical services [2].

For those diagnosed with non-metastatic prostate cancer, 5-year survival is nearly 100% after radiation, surgery, or conservative management (active surveillance or watchful waiting) [3,4]. Conservative management does not have the upfront costs associated with radiation and surgery, but it does require ongoing physician visits and testing which incur costs over time. To further complicate the tradeoffs associated with treatment choices, the enduring side effects associated with surgery or radiation frequently result in diminished quality-of-life and increased medical services compared to individuals undergoing conservative management [3,4].

Given the similarity in 5-year survival rates across non-metastatic prostate cancer treatment types [3,5], patients are confronted with a preference-sensitive treatment decision. This decision involves considering various treatment-related tradeoffs, including both financial implications and quality-of-life [4,6]. Clinicians are often interested in discussing care costs with their patients; however, the complexity and unknowns associated with treatment costs make these conversations difficult [7,8]

Both patients and clinicians encounter barriers to accessing information about treatment costs, which lies in direct contrast to the growing interest in providing transparent cost information through shared decision-making [6]. The integration of cost information into decision-making can support patients in their deliberations regarding the multitude of trade-offs inherent in prostate cancer treatment options.

This is particularly relevant for those with private health insurance. Of the almost 225,000 new prostate cancer diagnoses in the US in 2019, about one third (37%) of those cases were among men aged 45–64 years [9,10]. Those covered by private insurance often, have greater medical debt, spend more on care, and report that costs impact their access to care [11]. Individuals <65 years often experience variable costs; therefore, providing prostate cancer treatment cost estimates is highly applicable to this age group.

Our objective was to systematically quantify the cumulative total and patient out-of-pocket (OOP) costs linked to 3 non-metastatic prostate cancer treatment modalities from healthcare sector perspective, specifically: radiation, surgery, and conservative management at intervals of 1-, 3-, and 5-years post-diagnosis. We predicted cumulative total and OOP costs for a typical patient to improve cost transparency by quantifying cost information to facilitate conversations about potential costs and to advance cost evaluation research for non-metastatic prostate cancer.

## Materials and methods

### Data

Our retrospective cohort study sourced claims data from Merative™ MarketScan® Commercial Database. MarketScan Research databases include a nationally representative private insurance claims. We selected MarketScan Research databases due to its comprehensive coverage of privately insured individuals, including cost data related to cancer diagnoses and care needed for our analyses, which has been widely utilized for financial analyses in cancer [12–15]. We used MarketScan data from 2006–2020 (the most updated data at the time of study). The Washington University School of Medicine IRB determined this is non-human subjects research (IRB #200206084).

### Study design and patient population

#### Inclusion criteria.

We described our process for defining our cohort in prior work [16]. Briefly, we included all patients diagnosed with prostate cancer and with the absence of metastatic diseases using International Classification of Diseases, 9^th^/10^th^ Revision, Clinical Modification (ICD-9/10-CM). We included patients with at least 2 outpatient codes at least 30-days apart or 1 inpatient prostate cancer diagnosis (ICD-9 185, ICD-10 C61) from 2007−2020. We restricted the diagnosis period to 2007−2019 to ensure individuals diagnosed with prostate cancer had at least 1-year of data for identifying comorbidities and 1-year of follow-up data for cost analysis. Among these patients, the date of diagnosis (index date hereafter) was defined as the date of the first biopsy within +/- 30 days of a prostate cancer diagnosis, as the dates of biopsy and diagnosis may lag administratively.

We identified and used the patient’s initial treatment choice, determined by the first treatment codes present within 12-months following their index date. Based on their initial treatment choice following diagnosis, patients were categorized into 3 groups: 1) conservative management [3] (i.e., no surgery or radiation codes within 12-months of the index date), 2) surgery (i.e., open and laparoscopic radical prostatectomy), and 3) radiation (i.e., seeds/internal/brachytherapy and external beam radiotherapy). If a patient did not have treatment codes for either surgery or radiation within 12-months following their index date, the patient was considered to have selected conservative management.

#### Exclusion criteria.

Patients missing a clear date of diagnosis were excluded, along with individuals meeting any of the following criteria: 1) being female; 2) diagnosed with metastatic cancer within 12-months before or after the index date; 3) secondary cancer diagnosis other than prostate cancer within 12-months before or after the index date; 4) prostate cancer diagnosis within 11-months prior to the index date; 5) medical coverage for <12-months before the index date or less than X years after it (where X = 1, 3, or 5, representing the duration of the target cumulative cost of interest), indicating incomplete cost data; 6) age < 18 or >63 years at index, due to concerns about incomplete data related to Medicare eligibility; or 7) displaying missing or negative costs within the duration of the target cost due to administrative data entry errors. For 7), patients with negative or missing costs between Years 2 and 3 were excluded from Year 3 and patients with negative or missing costs between Years 3 and 5 were excluded from Year 5.

### Outcomes

We computed descriptive statistics for the patient sociodemographic characteristics of the full cohort and cumulative total costs from a healthcare sector perspective at 1-, 3-, and 5-years following their index date by summing all costs accumulated from index date to 1-, 3-, and 5-years (Tables 1 and 2). Total costs included the costs of all claims billed for the insurance provider for a patient (not including the costs for outpatient prescriptions). We also computed cumulative OOP costs. OOP costs included deductibles, copays, and coinsurance. Costs pooled from different years were adjusted to the 2020 price level based on the Consumer Price Index.

These costs were cumulative at 1-, 3-, and 5-years based on initial treatments (i.e., the presence of treatment-related codes surgery, radiation), therefore, if the patient transitioned to another treatment modality after 12-months, these costs incurred due to the transition were included to capture all costs associated with their initial treatment decision.

### Statistical analysis

To compare across treatment groups, we performed χ^2^ tests for categorical variables and ANOVA tests for continuous variables.

The outcomes (i.e., 1-, 3-, and 5-year total and OOP costs) were examined for the respective distribution via histograms. The plot showed that the outcomes are not normally distributed; therefore, all cost outcomes were log-transformed.

A multivariable regression model with inverse probability of treatment weighting (IPTW) was performed to account for the potential bias resulting from treatment selection (i.e., patients selected to receive a certain treatment incurred lower or higher cost due to disease severity, which by itself is associated with the incurred costs). The weights were generated by predicted propensity for each treatment based on fitting a multinomial logistic regression model with the same covariates mentioned below [17–19]. We used all covariates available in, or calculated from the MarketScan database, including age, year (to account for time trend), Charlson comorbidity score [20], region (northeast, north central, south, west, unknown), health plan (employer, health plan), plan type (comprehensive, EPO, HMO, POS, PPO, POS with capitation, CDHP, HDHP), and relation to employee (employee, spouse, child/other). Charlson comorbidity scores were computed based on all relevant diseases diagnoses via ICD codes within one year prior to the index date [20–22].

Based on the estimated regression, we predicted 1-, 3-, and 5-year total/OOP costs for a typical patient (using the mode for each covariate) in the respective analysis, if he underwent active surveillance, surgery, or radiation as his first treatment.

All statistical tests are two-sided. Statistical significance was assessed at the level of 0.05. All statistical analyses were performed using SAS 9.4.

## Results

### Cohorts

**Fig 1:**
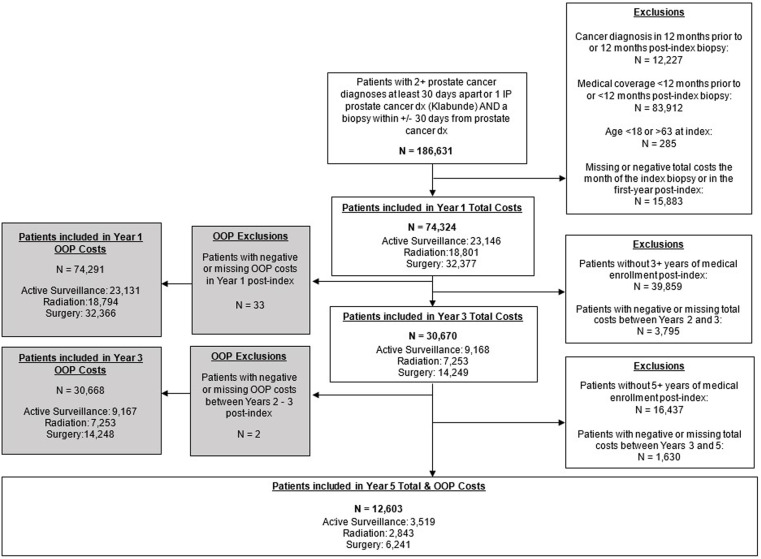
Consort diagram for the cumulative total costs & out-of- pocket costs for the full cohort.

Our full cohort included 74,324 patients in Year 1, 30,670 in Year 3 and 12,603 in Year 5. Years 3 and 5 are subsets of the Year 1 cohort (Fig 1).

For the OOP costs cohort, additional patients were excluded due to missing values (Table 1). Specifically, an additional 33 patients for Year 1 cohort (OOP/total cost cohort n = 74,291/74,324) and an additional 2 patients for the Year 3 cohort (30,668 OOP/total 30,670) were excluded from analysis due to negative or missing OOP costs. Year 5 was the same for both OOP and total cost cohorts (n = 12,603; Fig 1).

**Table 1:** Characteristics of the full cohort of privately insured men diagnosed with prostate cancer from 2007–2019 stratified by treatment group.

		Radiation	Surgery	Conservative Management	Total	p-value^*^
**N**		18,801	32,377	23,146	74,324	
**Age (years)**	**Mean (SD)**	58.29 (4.06)	56.69 (4.82)	57.42 (4.58)		<0.0001
**Charlson Comorbidity Score**	**Mean (SD)**	0.29 (0.75)	0.17 (0.53)	0.2 (0.60)		<0.0001
**Relation to Employee**		**n (%)**	**n (%)**	**n (%)**	**Total**	**p-value**
	**Employee**	14,729 (78.34)	25,525 (78.84)	17,916 (77.40)	58,170	0.0009
	**Spouse**	4,064 (21.62)	6,845 (21.14)	5,225 (22.57)	16,134	
	**Child/Other**	8 (0.04)	7 (0.02)	5 (0.02)	20	
**Health Plan Indicator** ^**‡**^						**p-value**
	**Employer**	11,488 (61.1)	19,995 (61.76)	14,636 (63.23)	46,119	<.0001
	**Health Plan**	7,313 (38.9)	12,382 (38.24)	8,510 (36.77)	28,205	
**Plan Type**		**n (%)**	**n (%)**	**n (%)**	**Total**	**p-value**
	**Comprehensive**	975 (5.28)	1342 (4.21)	960 (4.21)	3,277	<0.0001
	**EPO**	276 (1.50)	418 (1.31)	275 (1.21)	969	
	**HMO**	2,170 (11.76)	3,477 (10.91)	3,331 (14.61)	8,978	
	**POS**	1675 (9.08)	2,657 (8.34)	1,991 (8.73)	6,323	
	**PPO**	11,553 (62.61)	20,342 (63.81)	13,697 (60.07)	45,592	
	**POS w/ Capitation**	142 (0.77)	275 (0.86)	185 (0.81)	602	
	**CDHP**	1,193 (6.47)	2,270 (7.12)	1,545 (6.78)	5,008	
	**HDHP**	469 (2.54)	1,096 (3.44)	818 (3.59)	2,383	
**Region**		**n (%)**	**n (%)**	**n (%)**	**Total**	**p-value**
	**Northeast**	3,087 (16.42)	4,859 (15.01)	3,939 (17.02)	11,885	<0.0001
	**North Central**	4,292 (22.83)	8,675 (26.79)	5,150 (22.25)	18,117	
	**South**	8,554 (45.5)	14,149 (43.7)	9,393 (40.58)	32,096	
	**West**	2,634 (14.01)	4,274 (13.2)	4,358 (18.83)	11,266	
	**Unknown**	234 (1.24)	420 (1.3)	306 (1.32)	960	
**Year**		**n (%)**	**n (%)**	**n (%)**	**Total**	**p-value**
	**2007**	2,030 (10.8)	2,823 (8.72)	2,503 (10.81)	7,356	<0.0001
	**2008**	2,096 (11.15)	3,264 (10.08)	2,217 (9.58)	7,577	
	**2009**	2,343 (12.46)	3,888 (12.01)	2,459 (10.62)	8,690	
	**2010**	2,112 (11.23)	3,666 (11.32)	2,155 (9.31)	7,933	
	**2011**	2,171 (11.55)	4,090 (12.63)	2,353 (10.17)	8,614	
	**2012**	1,707 (9.08)	2,872 (8.87)	1,913 (8.26)	6,492	
	**2013**	1,481 (7.88)	2,617 (8.08)	1,800 (7.78)	5,898	
	**2014**	1,091 (5.8)	1,907 (5.89)	1,484 (6.41)	4,482	
	**2015**	1,061 (5.64)	2,022 (6.25)	1,522 (6.58)	4,605	
	**2016**	901 (4.79)	1,702 (5.26)	1,442 (6.23)	4,045	
	**2017**	867 (4.61)	1,612 (4.98)	1,482 (6.4)	3,961	
	**2018**	864 (4.60)	1,739 (5.37)	1,615 (6.98)	4,218	
	**2019**	77 (0.41)	175 (0.54)	201 (0.87)	453	

Note: conservative management treatment was defined as receipt of any other therapy other than surgery or radiation.

* P-value determined using chi-square test for categorical variables and Kruskal-Wallis test for age and cost variables.

‡ Health Plan Indicator: Whether the data supplier of the record was a large US employer or a Health Plan Percentages are based on column (treatment type) totals.

Abbreviations: SD: Standard Deviation; EPO: Exclusive Provider Organization; HMO: Health Maintenance Organization; POS: Point of Service; PPO: Preferred Provider Organization; CDHP: Consumer Driven Health Plan; HDHP: High Deductible Health Plan; OOP: Out-of-Pocket.

Most patients underwent surgery across the 3 time points (Year 1: 32,377, Year 3: 14,249, Year 5: 6,241). The second most common treatment type was conservative management (Year 1: 23,146, Year 3: 9,168, Year 5: 3,519), followed by radiation as the least common treatment type (Year 1: 18,801, Year 3: 7,253, Year 5: 2,843).

### Patient characteristics

For the cumulative total cost Year 1 full cohort (n = 74,324), patients were different across the 3 treatment groups in age (p < 0.0001), relation to employee (p = 0.0009), health plan indicator (p= < 0.0001), plan type (p < 0.0001), region (p < 0.0001), and year (p < 0.0001; Tables 1 and 2). For the Year 1 full cohort (n = 74,324), the mean ages across the 3 treatment groups were 57.4 (conservative management), 56.7 (surgery), and 58.3 (radiation) years (p < 0.0001). Most patients in the cohort had their data reported by their employer, were on an employer health plan, on PPO plans, living in the South region, and in year 2009. For the OOP Year 1 cohort (n = 74,291), the mean ages across the 3 treatment groups and other characteristics were significantly different and had the same majority characteristics as the full cohort.

**Table 2:** Total and OOP costs of the full cohort of privately insured men diagnosed with prostate cancer from 2007–2019 stratified by treatment group.

Costs (2020$)		Radiation		Surgery		Conservative Management		p-value*	
Total & OOP Costs		Total	OOP	Total	OOP	Total	OOP	Total	OOP
**Year 1**	**n**	18,801	18,794	32,377	32,366	23,146	23,131	--	--
	**Mean** **(SD)**	81,526 (36,790)	3,774 (10,743)	45,010 (61,752)	3,520 (16,632)	24,438 (27,948)	2,376 (2,944)	<0.0001	<0.0001
**Year 3**	**n**	7,253	7,253	14,249	14,248	9,168	9,167	--	--
	**Mean** **(SD)**	99,643 (78,325)	5,662 (5,037)	62,114 (50,792)	5,295 (4,226)	50,155 (52,860)	4,784 (4,329)	<0.0001	<0.0001
**Year 5**	**n**	2,843	2,843	6,241	6,241	3,519	3,519	--	--
	**Mean** **(SD)**	112,170 (79,304)	7,647 (7,026)	75,942 (68,237)	7,247 (6,018)	67,465 (66,705)	6,798 (5,504)	<0.0001	<0.0001

Note: conservative management treatment was defined as receipt of any other therapy other than surgery or radiation.

* P-value determined using Kruskal-Wallis test for cost variables.

‡ Abbreviations: SD: Standard Deviation; OOP: Out-of-Pocket.

Mean cumulative total costs for Year 1 were highest for radiation ($81,526), followed by surgery ($45,010) and conservative management ($24,438; p < 0.0001). Similarly for Year 3 cumulative total costs (radiation: $99,643 vs. surgery: $62,114 vs. conservative management: $50,155, p < 0.0001) and Year 5 cumulative total costs (radiation: $112,170 vs. surgery: $75,942 vs. conservative management: $67,465, p < 0.0001) were highest for radiation.

For the cumulative OOP cost Year 1 cohort (n = 74,291), mean OOP costs for Year 1 were highest for radiation ($3,774), followed by surgery ($3,520) and conservative management ($2,376; p < 0.0001). This was similar for cumulative OOP Year 3 costs (radiation: $5,662 vs. surgery: $5,295 vs. conservative management: $4,784, p < 0.0001) and for cumulative OOP Year 5 costs (radiation: $7,647 vs. surgery: $7,247 vs. conservative management: $6,798, p < 0.0001).

### Multivariable IPTW analysis

**Fig 2:**
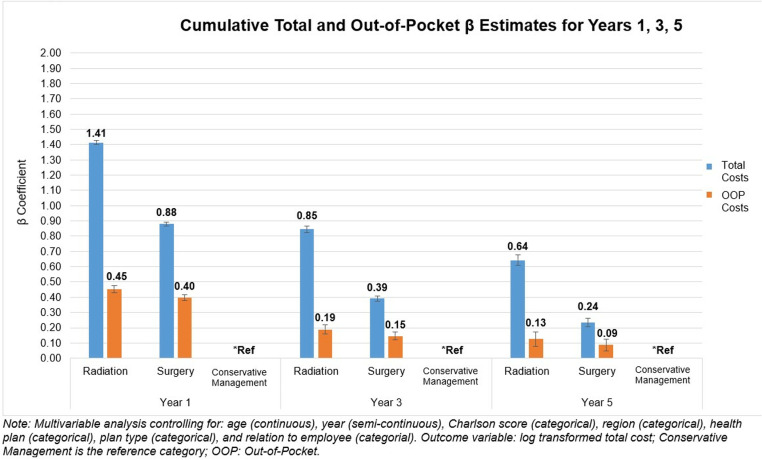
Multivariable IPTW analysis to compare cumulative total and out-of-pocket costs (in 2020$) across the 3 treatment groups at year 1, 3, and 5.

In the multivariable IPTW analysis, compared to conservative management, cumulative total cost was significantly higher for radiation at Years 1, 3, and 5, respectively (Year 1: β = 1.41, 95% Confidence Interval [CI]: 1.40–1.43, p < 0.0001; Year 3: β = 0.85, 95% CI: 0.82–0.87, p < 0.0001; Year 5: β = 0.64, 95% CI: 0.61–0.68, p < 0.0001; Fig 2, see Table 1A in S1 File for the βs of all included covariates in the analysis). Cumulative total cost for surgery was also higher than conservative management at Years 1, 3, and 5 (Year 1: β = 0.88, 95% CI: 0.87–0.89, p < 0.0001; Year 3: β = 0.39, 95% CI: 0.37–0.41, p < 0.0001; Year 5: β = 0.24, 95% CI: 0.21–0.26, p < 0.0001).

This was similar for cumulative OOP cost with radiation significantly higher at Years 1/3/5 (Year 1: β = 0.45, 95% CI: 0.43–0.48, p < 0.0001; Year 3: β = 0.19, 95% CI: 0.16–0.22, p < 0.0001; Year 5: β = 0.13, 95% CI: 0.08–0.17, p < 0.0001; Fig 2, see Table 1B in S1 File for the βs of all included covariates in the analysis). Cumulative OOP cost for surgery was also higher than conservative management at years 1, 3, and 5, respectively (Year 1: β = 0.40, 95% CI: 0.38–0.42, p < 0.0001; Year 3: β = 0.15, 95% CI: 0.12–0.17, p < 0.0001; Year 5: β = 0.09, 95% CI: 0.05–0.12, p < 0.0001).

### Predicted total costs

**Fig 3:**
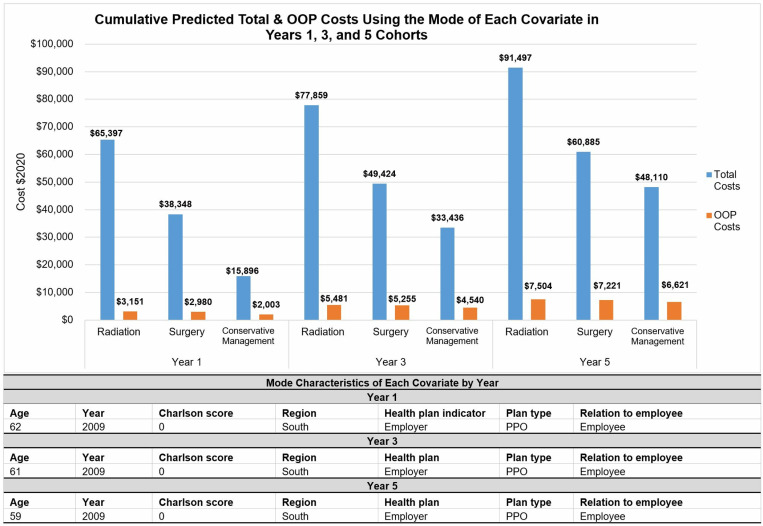
Predicted cumulative total and out-of-pocket costs (in 2020$) for patients with the mode characteristics of each covariate in years 1, 3, and 5 cohorts.

For the 1-year outcome, the typical (mode) patient is diagnosed at 62 years with a Charlson Comorbidity Score of 0 and a PPO health plan in the South region in 2009, the cumulative total cost at Year 1 for conservative management was $15,896, for surgery was $38,348, and for radiation was $65,397 (Fig 3). Year 1 cumulative OOP costs for conservative management was $2,003, for surgery was $2,980, and for radiation was $3,151.

For the 3-year outcome, the typical (mode) patient is diagnosed at 61 years with a Charlson Comorbidity Score of 0 and a PPO health plan in the South region in 2009, the cumulative total cost at year 3 if he received conservative management was $33,436, if he received surgery was $49,424, and he received radiation was $77,859. Year 3 cumulative OOP for conservative management was $4,540, for surgery was $5,255, and for radiation was $5,481.

For the 5-year outcome, the typical (mode) patient is diagnosed at 59 years with a Charlson Comorbidity Score of 0 and a PPO health plan in the South region in 2009, the cumulative total cost at Year 5 if he received conservative management was $48,110, for surgery was $60,885, and he received radiation was $91,497. Year 5 cumulative OOP costs for conservative management was $6,621, for surgery was $7,221, and for radiation was $7,504.

## Discussion

We analyzed Year 1, 3, and 5 total and OOP costs following a diagnosis of prostate cancer in >70,000 privately insured men between 2007 and 2019. For all years, the cumulative costs of radiation were highest, followed by surgery and conservative management, respectively. We also predicted Year 1, 3, and 5 cumulative total and OOP costs for a typical patient to help improve cost transparency and facilitate cost conversations for non-metastatic prostate cancer. We found that radiation as first treatment had higher costs compared to surgery and conservative management at 3 time points.

Our findings extend extant literature suggesting conservative management typically has lower total costs when compared to definitive treatment, including surgery and radiation [23–25]. However, prior research shows that specific types of radiation (i.e., external beam) and surgery (e.g., robotic-assisted radical proctectomy) often exceed the costs of radiation using brachytherapy and surgery using open or laparoscopic prostatectomy [26,27]. Methods used to estimate costs vary, whether they are up front costs or costs over time, as well as the source of data for these estimates (e.g., national, local, Medicare, Medicaid, private). We add to this body of literature by using national private insurance claims data and estimating costs at 1-,3-, and 5-years showing that contemporary conservative management protocols do incur total costs up front and over time, with cumulative total costs nearing the cost of surgery in Year 5. These estimates are essential source data when conducting decision and cost-effective analyses.

OOP cost estimates help provide transparency to the direct costs associated with non-metastatic prostate cancer treatment, an increasingly important part of cancer care in the pursuit of addressing financial harm to improve patient outcomes. The majority of those in the cohort were on employer-sponsored insurance. Those undergoing cancer treatment often experience changes in their employment status, whether that involves lowering the number of hours worked, seeking less demanding employment, or ending their employment entirely [28,29]. Even with employer-sponsored insurance, patients in the US incur high OOP costs [30]. We found in Year 5 that patients incurred cumulative OOP costs of $7,504 for radiation, $7,221 for surgery, and $6,621 for conservative management. In this evolving employment context, patients may greatly benefit from knowing the varying OOP costs associated with treatment options and may incorporate those earlier into their decision making to help prepare for costs associated with their care.

Health insurance plan type, including health maintenance organizations (HMOs), high deductible health plans (HDHPs), preferred provider organizations (PPOs) and other plans, offer differing coverage and payment structures. For example, PPOs typically have higher monthly premiums compared to HMOs with the option to seek caree from out-of-network providers [31]. However, if receiving care from an out-of-network provider, OOP fees may not be included in annual out-of-pocket maximums, potentially exposing enrollees to unanticipated costs [31]. Those enrolled in HDHPs are also susceptible to financial harm, particularly those with lower income [32]. This financial strain is even more pronounced among individuals with cancer, as the costs typically surpass those of other chronic illnesses [33]. Even within the broader categories of health plans, individuals’ insurance plans are highly variable and caution should be applied when estimating costs.

MarketScan data has been used to estimate costs for multiple types of cancer care [13], costs for metastatic prostate cancer [15], costs in the context of metastatic castration-sensitive prostate cancer [34,35], total and OOP costs for hormone sensitive-prostate cancer [36], among other cost estimates across different health conditions. Using commercial claims data is important because financial harm is a serious issue for cancer patients, especially for those not eligible for Medicare. Among cancer survivors, those 18–64 years are more likely to experience financial harm compared to those ≥65 years [37]. The lack of cost transparency associated with cancer care can bring forth unanticipated costs without the option to prepare. These financial demands contribute to patients' medical debt, exacerbate psychological stressors, and can lead to diminished health outcomes [38–40]. To ameliorate the excessive stress associated with the financial costs of care, providing transparency to prepare and connect patients with financial resources earlier in the treatment process can help give patients and their families an opportunity to avert the unanticipated challenges associated with financial hardship [6].

### Limitations

MarketScan data lacks PSA, Gleason score, and tumor staging data. This limitation restricted our ability to define low-risk prostate cancer, although we excluded patients with a code of metastatic disease. Estimated costs by treatment types may vary by specific tumor type, score, or stage. Nonetheless, the primary goal of the analysis is to enhance understanding of non-metastatic prostate cancer treatment-specific costs. Also, MarketScan databases only encompass claims data for insured individuals, thereby excluding the uninsured from our analysis. Additionally, the absence of race or ethnicity data means our analysis cannot adequately explore the impact of these factors on costs. Active surveillance and watchful waiting are distinct treatment approaches; however, billing records may appear identical. Therefore, our research team used a 12-month timeframe to suggest a patient’s selection of conservative management. Patients who chose conservative management could transition to other treatment types, which would be captured in the accumulated total cost in the corresponding years. Yet, the purpose of this study was to provide cost estimates to summarize the total cost, which include the possibility of future transition to other treatments. While 1-, 3-, and 5-year cumulative costs are an important contribution, costs associated with longer-term sequala may occur outside of that timeframe. In the future, conducting a decision analysis analyzing the potential treatment sequence and longer-term sequala could advance this work.

### Strengths and implications

Our estimate of non-metastatic prostate cancer treatment costs among privately insured individuals is an important step toward providing cost transparency to those making treatment decisions. Some clinicians might be guiding treatment based on clinical characteristics, while some might be engaging patients in shared discussions about multiple possible options. When there are choices, cost transparency is important to help people consider costs alongside other tradeoffs particularly when 5-year survival is similar for non-metastatic prostate cancer across the 3 treatment options [3–5]. This can allow clinicians and patients to weigh their treatment options with additional information that can help them prepare for potential costs associated with their care and to avoid being surprised which can contribute to financial burden. This analysis is not designed to persuade patients and clinicians to recommend one treatment over another; rather, it is an additional attribute to include when weighing treatment options.

## Conclusions

Radiation as a first treatment option had higher costs compared to surgery and conservative management at all 3 time points, respectively. These cost estimates have the potential to help researchers as they evaluate costs for non-metastatic prostate cancer and to advance the translation of anticipated costs to help guide patients and clinicians as they discuss treatment options.

## Supporting information

S1 File **Table 1A.** Multivariable IPTW analysis results: Total Costs. **Table 1B.** Multivariable IPTW analysis results: OOP Costs.
(DOCX)
