# Supplementary material for: Costs of non-metastatic prostate cancer treatment among privately insured men in the United States
Source: PLoS One. 2025 May 30;20(5):e0324902. doi: 10.1371/journal.pone.0324902 (PMC12124492; doi:10.1371/journal.pone.0324902)
Supplement: S1 File — Table 1A. Multivariable IPTW analysis results: Total Costs. Table 1B. Multivariable IPTW analysis results: OOP Costs. (DOCX) [file pone.0324902.s001.docx]

| **Supplementary Table 1A.** **Multivariable IPTW analysis results: Total Costs.** | | | | | | |
| --- | --- | --- | --- | --- | --- | --- |
|  | **Year 1** | | **Year 3** | | **Year 5** | |
|  | **β (95% CI)** | **p-value*** | **β (95% CI)** | **p-value*** | **β (95% CI)** | **p-value*** |
| **Year** | -0.0215 (-0.0232, -0.0198) | <.0001 | -0.0074 (-0.0104, -0.0044) | <.0001 | -0.0044 (-0.0099, 0.0010) | 0.1093 |
| **Age (years)** | 0.0018 (0.0007, 0.0030) | 0.0018 | 0.0053 (0.0034,0.0072) | <.0001 | 0.0101 (0.0070, 0.0132) | <.0001 |
| **Charlson** | 0.0662 (0.0578, 0.0746) | <.0001 | 0.1068 (0.0927, 0.1210) | <.0001 | 0.1105 (0.0881, 0.1328) | <.0001 |
| **Relation to Employee** | | | | | | |
| **Employee** | 0.1057 (-0.2203, 0.4317) | 0.5251 | 0.0569 (-0.4366, 0.5503) | 0.8213 | 0.1464 (-0.5602, 0.8530) | 0.6846 |
| **Spouse** | 0.0959 (-0.2303, 0.4220) | 0.5646 | 0.0522 (-0.4416, 0.5459) | 0.8360 | 0.1400 (-0.5671, 0.8470) | 0.6980 |
| **Child/Other** | 0.0000 (0.0000, 0.0000) | -- | 0.0000 (0.0000, 0.0000) | -- | 0.0000 (0.0000, 0.0000) | -- |
| **Health Plan Indicator^‡^** | | | | | | |
| **Employer** | -0.0369 (-0.0485, -0.0252) | <.0001 | -0.0471 (-0.0657, -0.0286) | <.0001 | -0.0467 (-0.0785, -0.0148) | 0.0041 |
| **Health Plan** | 0.0000 (0.0000, 0.0000) | -- | 0.0000 (0.0000, 0.0000) | -- | 0.0000 (0.0000, 0.0000) | -- |
| **Plan Type** | | | | | | |
| **Comprehensive** | -0.0538 (-0.0930, -0.0147) | 0.0070 | -0.0437 (-0.1064, 0.0190) | 0.1716 | -0.0605 (-0.1642, 0.0433) | 0.2533 |
| **EPO** | -0.0280 (-0.0828, 0.0268) | 0.3160 | 0.0229 (-0.0695, 0.1152) | 0.6273 | 0.0363 (-0.1225, 0.1951) | 0.6545 |
| **HMO** | -0.0993 (-0.1328, -0.0657) | <.0001 | -0.0769 (-0.1324, -0.0214) | 0.0066 | -0.0356 (-0.1299, 0.0586) | 0.4588 |
| **POS** | -0.0149 (-0.0500, 0.0203) | 0.4077 | -0.0022 (-0.0599, 0.0555) | 0.9404 | -0.0143 (-0.1108, 0.0823) | 0.7724 |
| **PPO** | -0.0068 (-0.0375, 0.0239) | 0.6657 | -0.0042 (-0.0565, 0.0481) | 0.8751 | 0.0086 (-0.0822, 0.0993) | 0.8535 |
| **POS w/ Capitation** | -0.1336 (-0.1996, -0.0676) | <.0001 | -0.2026 (-0.3135, -0.0917) | 0.0003 | -0.1943 (-0.3899, 0.0012) | 0.0514 |
| **CDHP** | -0.0718 (-0.1076, -0.0360) | <.0001 | -0.0643 (-0.1229, -0.0058) | 0.0313 | -0.0711 (-0.1703, 0.0282) | 0.1604 |
| **HDHP** | 0.0000 (0.0000, 0.0000) | -- | 0.0000 (0.0000, 0.0000) | -- | 0.0000 (0.0000, 0.0000) | -- |
| **Region** | | | | | | |
| **Northeast** | 0.0928 (0.0441, 0.1416) | 0.0002 | 0.1037 (0.0211, 0.1862) | 0.0138 | 0.2640 (0.0946, 0.4334) | 0.0023 |
| **North Central** | 0.0542 (0.0061, 0.1024) | 0.0273 | 0.0518 (-0.0300, 0.1335) | 0.2147 | 0.1973 (0.0289, 0.3656) | 0.0216 |
| **South** | -0.0319 (-0.0795, 0.0156) | 0.1883 | -0.0203 (-0.1012, 0.0606) | 0.6229 | 0.1430 (-0.0241, 0.3101) | 0.0935 |
| **West** | 0.0877 (0.0387, 0.1366) | 0.0004 | 0.1263 (0.0435, 0.2090) | 0.0028 | 0.3021 (0.1323, 0.4719) | 0.0005 |
| **Unknown** | 0.0000 (0.0000, 0.0000) | -- | 0.0000 (0.0000, 0.0000) | -- | 0.0000 (0.0000, 0.0000) | -- |
| *Note: conservative management treatment was defined as receipt of any other therapy other than surgery or radiation.*  **P-value determined using chi-square test for categorical variables and Kruskal-Wallis test for age and cost variables.*  *‡Health Plan Indicator: Whether the data supplier of the record was a large US employer or a Health Plan Percentages are based on column (treatment type) totals.*  *Abbreviations: SD: Standard Deviation**; CI: Confidence Interval; Charlson: Charlson Comorbidity Score; EPO: Exclusive Provider Organization; HMO: Health Maintenance Organization; POS: Point of Service; PPO: Preferred Provider Organization; CDHP: Consumer Driven Health Plan; HDHP: High Deductible Health Plan; OOP: Out-of-Pocket.* | | | | | | |

| **Supplementary Table 1B.** **Multivariable IPTW analysis results: OOP Costs.** | | | | | | |
| --- | --- | --- | --- | --- | --- | --- |
|  | **Year 1** | | **Year 3** | | **Year 5** | |
|  | **β (95% CI)** | **p-value*** | **β (95% CI)** | **p-value*** | **β (95% CI)** | **p-value*** |
| **Year** | -0.0065 (0.0038, 0.0092) | <.0001 | -0.0000 (-0.0042, 0.0042) | 0.9953 | 0.0031 (-0.0042, 0.0104) | 0.4075 |
| **Age (years)** | 0.0006 (-0.0024, 0.0013) | 0.5561 | 0.0043 (0.0016, 0.0069) | 0.0017 | 0.0080 (0.0038, 0.0121) | 0.0002 |
| **Charlson** | -0.0186 (-0.0320, -0.0051) | 0.0069 | 0.0478 (0.0277, 0.0680) | <.0001 | 0.0860 (0.0559, 0.1161) | <.0001 |
| **Relation to Employee** | | | | | | |
| **Employee** | 0.9667 (0.4440, 1.4895) | 0.0003 | 1.2605 (0.5593, 1.9616) | 0.0004 | 0.9830 (0.0313, 1.9347) | 0.6846 |
| **Spouse** | 0.8683 (0.3452, 1.3913) | 0.0011 | 1.1870 (0.4855, 1.8886) | 0.0009 | 0.9433 (-0.0090, 1.8956) | 0.6980 |
| **Child/Other** | 0.0000 (0.0000, 0.0000) | -- | 0.0000 (0.0000, 0.0000) | -- | 0.0000 (0.0000, 0.0000) | -- |
| **Health Plan Indicator^‡^** | | | | | | |
| **Employer** | -0.0494 (-0.0681, -0.0307) | <.0001 | -0.0060 (-0.0324, 0.0203) | 0.6536 | 0.0268 (-0.0161, 0.0697) | 0.2203 |
| **Health Plan** | 0.0000 (0.0000, 0.0000) | -- | 0.0000 (0.0000, 0.0000) | -- | 0.0000 (0.0000, 0.0000) | -- |
| **Plan Type** | | | | | | |
| **Comprehensive** | -0.9749 (-1.0377, -0.9122) | <.0001 | -0.8665 (-0.9556, -0.7774) | <.0001 | -0.8778 (-1.0175, -0.7381) | <.0001 |
| **EPO** | -1.2240 (-1.3119, -1.1361) | <.0001 | -1.2339 ( -1.3651, -1.1027) | <.0001 | -1.0743 (-1.2882, -0.8604) | <.0001 |
| **HMO** | -1.8786 (-1.9324, -1.8248) | <.0001 | -1.7741 (-1.8529, -1.6953) | <.0001 | -1.5854 (-1.7124, -1.4584) | <.0001 |
| **POS** | -1.1268 (-1.1832, -1.0703) | <.0001 | -1.0822 ( -1.1641, -1.0003) | <.0001 | -1.0626 (-1.1927, -0.9325) | <.0001 |
| **PPO** | -0.6769 (-0.7262, -0.6277) | <.0001 | -0.6910 (-0.7654, -0.6167) | <.0001 | -0.6949 (-0.8171, -0.5727) | <.0001 |
| **POS w/ Capitation** | -1.7395 (-1.8453, -1.6336) | <.0001 | -1.7754 (-1.9329, -1.6178) | <.0001 | -1.6118 (-1.8752, -1.3484) | <.0001 |
| **CDHP** | -0.3256 (-0.3831, -0.2681) | <.0001 | -0.2907 (-0.3739, -0.2075) | <.0001 | -0.2923 (-0.4260, -0.1586) | <.0001 |
| **HDHP** | 0.0000 | -- | 0.0000 (0.0000, 0.0000) | -- | 0.0000 (0.0000, 0.0000) | -- |
| **Region** | | | | | | |
| **Northeast** | -0.3465 (-0.4247, -0.2684) | <.0001 | -0.2322 (-0.3494, -0.1149) | 0.0001 | 0.0003 (-0.2278, 0.2285) | 0.9978 |
| **North Central** | -0.0388 (-0.1160, 0.0385) | 0.3252 | 0.0009 (-0.1153, 0.1171) | 0.9874 | 0.1282 (-0.0985, 0.3550) | 0.2677 |
| **South** | 0.2596 (0.1833, 0.3358) | <.0001 | 0.2645 (0.1495, 0.3795) | <.0001 | 0.3899 (0.1649, 0.6150) | 0.0007 |
| **West** | -0.0584 (-0.1369, 0.0201) | 0.1448 | -0.0240 (-0.1416, 0.0937) | 0.6895 | 0.0805 (-0.1482, 0.3092) | 0.4903 |
| **Unknown** | 0.0000 (0.0000, 0.0000) | -- | 0.0000 (0.0000, 0.0000) | -- | 0.0000 (0.0000, 0.0000) | -- |
| *Note: conservative management treatment was defined as receipt of any other therapy other than surgery or radiation.*  **P-value determined using chi-square test for categorical variables and Kruskal-Wallis test for age and cost variables.*  *‡Health Plan Indicator: Whether the data supplier of the record was a large US employer or a Health Plan Percentages are based on column (treatment type) totals.*  *Abbreviations: SD: Standard Deviation; CI: Confidence Interval; Charlson: Charlson Comorbidity Score; EPO: Exclusive Provider Organization; HMO: Health Maintenance Organization; POS: Point of Service; PPO: Preferred Provider Organization; CDHP: Consumer Driven Health Plan; HDHP: High Deductible Health Plan; OOP: Out-of-Pocket.* | | | | | | |
